# Bioaccessibility of Antioxidants in Prickly Pear Fruits Treated with High Hydrostatic Pressure: An Application for Healthier Foods

**DOI:** 10.3390/molecules26175252

**Published:** 2021-08-30

**Authors:** Andrea Gómez-Maqueo, Dora Steurer, Jorge Welti-Chanes, M. Pilar Cano

**Affiliations:** 1 Biotechnology and Microbiology of Food Department, Institute of Food Science Research (CIAL, CSIC-UAM), Nicolás Cabrera 9, 28049 Madrid, Spain; agmaqueo@gmail.com (A.G.-M.); dora.steurer@gmail.com (D.S.); 2 Tecnologico de Monterrey, Escuela de Ingeniería y Ciencias, Ave. Eugenio Garza Sada 2501, Monterrey 64700, Mexico; jwelti@tec.mx; 3 Food Structure Team, Clinical Nutrition Research Center, Singapore Institute of Food and Biotechnology Innovation, Agency for Science, Research and Technology, 14 Medical Drive #07–02, MD 6 Building, Yong Loo Lin School of Medicine, Singapore 117599, Singapore

**Keywords:** *Opuntia ficus-indica*, high hydrostatic pressure, bioaccessibility, digestive stability, betalains, phenolic compounds

## Abstract

High hydrostatic pressure (HHP) is a commercial processing technology which can enhance the health potential of foods by improving the bioaccessibility of their bioactive compounds. Our aim was to study the bioaccessibility and digestive stability of phenolic compounds and betalains in prickly pear fruits (*Opuntia ficus-indica* L. Mill. var. Pelota and Sanguinos) treated with HHP (100, 350, and 600 MPa; come-up time and 5 min). The effects of HHP on pulps (edible fraction) and peels (sources of potential healthy ingredients) were assessed. In pulps, betanin bioaccessibility increased (+47% to +64%) when treated at 350 MPa/5 min. In HHP-treated pulps, increases in the bioaccessibility of piscidic acid (+67% to +176%) and 4-hydroxybenzoic acid glycoside (+126% to 136%) were also observed. Isorhamnetin glycosides in peels treated at 600 MPa/CUT had higher bioaccessibility (+17% to +126%) than their controls. The effects of HHP on the bioaccessibility of health-promoting compounds are not exclusively governed by extractability increases of antioxidants in the food matrix (direct effects). In this work we found evidence that indirect effects (effects on the food matrix) could also play a role in the increased bioaccessibility of antioxidants in fruits treated with HHP.

## 1. Introduction

Prickly pear (*Opuntia ficus-indica* L. Mill.) fruits from the Cactaceae family represent nutritious sources of healthy foods adaptable to expanding regions of hot climate. Prickly pears are the most widely consumed fruits from the plants of the *Opuntia* genus and are cultivated in Latin America, Africa, and the Mediterranean region. They can be found in Mexico and in Spain, where colored varieties such as Pelota and Sanguinos are widely cultivated. Prickly pear fruits may be commercialized as fresh fruits or as derived products such as juices and jams. During the preparation of *Opuntia* beverages, a large amount of waste and by-products are produced mainly from the peels of the fruits. Prickly pear peels could be sustainable sources of nutrients and bioactive compounds. Moreover, the recovery of high-added value compounds from *Opuntia* waste and by-products provides dual benefits by addressing both management of bio-waste and societal health [1].

Betalains and phenolic compounds are health-promoting compounds which contribute greatly to the health potential of colored prickly pear varieties. Red-colored betanin and yellow-colored indicaxanthin are the most abundant betalains in these fruits and are responsible for their free radical scavenging and antioxidant activity [2]. Prickly pears are also abundant in phenolic acids such as piscidic acid, which is also found in other members of the Cactaceae family. Piscidic acid, despite being less studied compared to other phenolic compounds, has shown anti-hypercholesterolemia effects by inhibiting cholesterol permeation in vitro as well as anti-inflammatory activity [3,4]. Furthermore, prickly pears are rich sources of flavonoids such as isorhamnetin glycosides, which possess significant antioxidant and anti-inflammatory activities [5].

For betalains and phenolic compounds to be able to exert the mentioned health benefits in vivo, they must be bioaccessible. The bioaccessibility of a bioactive compound refers to the fraction which is released from the food matrix, modified in the gastrointestinal tract, and that is available for potential absorption [6]. This includes the transformations to the food matrix, which occur during digestion, absorption by epithelial cells in the intestine, and finally presistemic metabolism (intestinal and hepatic). It is possible to assess digestive stability and bioaccessibility via standardized static in vitro simulated gastrointestinal digestion methodologies such as the INFOGEST methodology [7] as used in this study.

High hydrostatic pressure (HHP) is a non-thermal technology traditionally used to assure microbiological safety in foods, meanwhile preserving their sensorial characteristics. However, in the last years there has been significant progress made in the use of HHP for promoting healthy attributes in foods. Some potential applications being explored include enhancing or retaining nutritional value, retaining immunoglobulin components in dairy products, increasing resistant starch content in cereals, reducing glycemic index of fruits, and promoting the extraction of bioactive compounds from food waste [8]. This last application has been widely studied in the last years. However, the underlying principles and mechanisms are not yet fully understood and more studies are needed to optimize HHP treatments on different bioactive compounds in food products with important effects on health, opening doors to new HHP applications in the food industry [9].

We recently studied the microstructural changes in prickly pear cells submitted to HHP treatments to identify the mechanisms by which bioactive compounds could be released from their intracellular compartments [10]. This was done to gain more information on the effect of HHP on bioactive extractability and in vitro antioxidant and anti-inflammatory activities in prickly pear fruits [11]. This last study focused on the direct and quantifiable changes of bioactive compound concentration after pressurization. Although insightful, the contribution of high hydrostatic pressure to producing healthier foods should be further substantiated by evidence on the digestive stability and bioaccessibility of their bioactive compounds. Furthermore, confirmation is needed to see if the HHP treatments that result in the highest release of health promoting compounds (direct effect) are, in fact, the most bioaccessible; or if, contrarily, a premature release of bioactive compounds could have a negative effect on their bioaccessibility.

Moreover, studies on the digestive stability of health-promoting compounds during each phase of simulated gastrointestinal digestion can be useful for elucidating other effects of HHP on the bioaccessibility of bioactive compounds (indirect effects). Beneficial indirect effects caused by HHP can include (i) microstructural changes to the food matrix that can promote the release of phytochemicals during digestion because of a higher exposure to enzymes from the digestive tract, (ii) changes in viscosity due to modification of proteins or pectins that can have a protective effect on bioactive compounds from digestive conditions (i.e., pH shifts, enzymes) and even delay their release, and (iii) the activation/inactivation of endogenous enzymes to the food matrix. However, the use of HHP for enhancing the bioaccessibility of bioactive compounds should be considered a double-edge sword since the mentioned changes to the food matrix can also be detrimental to bioactive compound bioaccessibility under certain conditions.

Hence, the aim of this work was to study the changes in digestive stability and bioaccessibility of individual phenolic compounds and betalains in prickly pear fruits (*Opuntia ficus-indica* L. Mill. Var. Pelota and Sanguinos) treated with HHP (100, 350, and 600 MPa; come-up time and 5 min) and in untreated fruits (control samples). With this study we expect to contribute to the knowledge of the use of HHP treatment for promoting the health potential of foods by enhancing the bioaccessibility of their bioactive compounds, which is a limiting factor for their potential in vivo bioactivities.

## 2. Results and Discussion

### 2.1. Main Bioactive Compounds in Pelota and Sanguinos Prickly Pear Fruits

The main bioactive compounds in control and pressurized Pelota and Sanguinos peels and pulps are shown in Table 1. The main betalains (betanin and indicaxanthin), phenolic acids (piscidic acid and 4-hydroxybenzoic acid glycoside), and flavonoids (isorhamnetin glycosides) were quantified. The complete betalain and phenolic profile in Sanguinos and Pelota prickly pear fruit varieties was reported in a previous study [12], where besides the main bioactive compounds shown in the present work, other betalains (portulacaxanthin, vulgaxanthin, betanidin) and phenolic compounds (quercetin glycosides and kaempferol glycosides) were found in minor concentrations.

As shown in Table 1, the red-colored betanin is the most abundant betalain in red-colored Sanguinos and purple-colored Pelota prickly pear tissues. The Pelota variety has 12.6 and 1.8 times more betanin in pulps and peels, respectively, compared to the Sanguinos prickly pear fruit. Yellow colored indicaxanthin was also found in peels and pulps of both prickly pear fruit varieties. The Pelota variety had 3.4 times higher indicaxanthin content in pulps and 0.6 times higher indicaxanthin content in peels than Sanguinos ones.

Besides betalains, prickly pear pulps contained piscidic acid and 4-hydroxybenzoic acid glycoside, being again the Pelota fruits that contain higher content in phenolic acids. In both varieties, peel tissue contained from 3 to 6.5 times higher piscidic acid and from 5.3 to 15.3 times higher 4-hydroxybenzoic acid glycoside than pulp tissue.

Meanwhile, the isorhamnetin glycosides were only detected in prickly pear peels (Table 1). The most abundant flavonoids were, namely, isorhamnetin glucosyl-rhamnosyl-rhamnoside (IG1), isorhamnetin glucosyl-rhamnosyl-pentoside (IG2), isorhamnetin hexosyl-hexoyl-pentoside (IG3), isorhamnetin glucosyl-pentoside (IG4), and isorhamnetin glucosyl-rhanoside (IG5). Purple-colored Pelota prickly pear fruits have a higher IG1 and IG5 content, whereas red-colored prickly pear fruits such as Sanguinos are characterized by a higher IG2 and IG5 profile. Total isorhamnetin glycoside concentration in peels was 6.28 and 4.43 mg/100 g fresh weight in Sanguinos and Pelota prickly pear peels.

### 2.2. Effect of HPP-Treatment on Individual Phenolic and Betalain Compounds Content

The content of individual betalain and phenolic compounds in control and pressurized (100, 350, and 600 MPa; CUT & 5 min) Sanguinos and Pelota prickly pear tissues (pulp and peel) after HHP-treatment were investigated in a previous study [11].

### 2.3. Bioaccessibility of Betalains and Phenolic Compounds in HHP-Treated Pulps

Increases in the bioaccessibility of bioactive compounds in prickly pear tissues (peels and pulps) due to HHP treatments may be attributed to direct and/or indirect effects. A direct effect of HHP refers to the increase in the extractability of the bioactive compound observed post-HHP-treatment. An indirect effect of HHP includes any changes to the food matrix and changes in other main components of the fruit tissues, which could promote the bioaccessibility of their bioactive compounds by enhancing their digestive stability.

The bioaccessibility of betalains and phenolic compounds in non-treated prickly pear pulps (control samples), and pressurized ones (100, 350, and 600 MPa; CUT and 5 min) are shown in Table 2. Graphical representation of this data may be consulted in Supplementary Figure S1.

The bioaccessibility of indicaxanthin (Table 2) in all prickly pear pulps treated with HHP was similar or lower than in their respective control samples (*p* ≤ 0.05). HPP treatment at 350 MPa/CUT and 350 MPa/5 min did not affect the bioaccessibility of indicaxanthin. However, HPP treatments such as 600 MPa/CUT, 100 MPa/5 min, and 350 MPa/5 min significantly (*p* ≤ 0.05) reduced the bioaccessibility of indicaxanthin in pulps of both prickly pear varieties. In Sanguinos pulps, bioaccessibility reductions of −53%, −45%, and −58% could be observed after the mentioned HPP treatments. This low bioaccessibility of indicaxanthin in pressurized pulps is most likely attributed to the changes in the components present in the food matrix as enzymes and polysaccharides among others (indirect effects of pressure).

On the other hand, betanin was more bioaccessible in Sanguinos and Pelota pulps treated at 350 MPa/5 min than in their respective controls (Table 2). After a HPP treatment at 350 MPa/5 min, the bioaccessibility of betanin in Pelota and Sanguinos pulps was 70% and 66%, compared to 42% and 45% in untreated pulps, respectively. Despite conserving betanin content post-pressurization, other HHP treatments caused negative effects (decreases) in the betanin bioaccessibility. Similar to what was observed for indicaxanthin, the bioaccessibility of betanin in pressurized pulps was mostly affected by changes to the food matrix caused by pressurization (indirect effects), rather than by degradation of the mentioned betalains during treatment (direct effects).

Contrarily, the bioaccessibility of phenolic acids in prickly pear pulps treated with high hydrostatic pressure was enhanced by most treatments (Table 2). Sanguinos and Pelota pressurized pulps processed at 350 MPa/5 min showed +68% and +179% higher bioaccessibility of piscidic acid, respectively, compared to their controls. Pulps processed at 100 MPa/5 min also showed a higher bioaccessibility of piscidic acid than their respective controls. In this case, the enhanced extractability of piscidic acid (direct effect) observed after treatment in pressurized prickly pear pulps was a main factor that strongly contributed to its higher bioaccessibility. Similarly, researchers [13] studied the effect of HHP on phenolic content in fruit juices and suggested that the phenols linked to the food matrix are released during pressurization, improving their extractability and therefore their content. Furthermore, these authors found 38% higher bioaccessibility of phenolic acids (caffeic, p-coumaric, chlorogenic, and ferulic) in pressurized juice-based beverages.

Meanwhile, 4-hydroxybenzoic acid glycoside showed lower bioaccessibility (Table 2) compared to other bioactive phenolic compounds. In untreated Sanguinos and Pelota pulps (control samples) its bioaccessibility was 20% and 17%, respectively, which is in agreement to what has been reported in orange-, red-, and white-colored prickly pear pulps from the Canary Islands [14]. The best treatments were at 100 MPa/CUT (+45% to +53% higher bioaccessibility) and 350/5 min (+135% to +124% higher bioaccessibility). These treatments have previously shown to enhance phenolic acid extractability in prickly pear pulps [11].

Processing at 350 MPa/5 min was the best treatment for increasing the extractability of betanin, piscidic acid, and 4-hydroxybenzoic acid glycoside in the pulps of both prickly pear varieties. Hence, the digestive stability of these samples during each stage of digestion was further studied and discussed in Section 2.5.1.

### 2.4. Bioaccessibility of Betalains and Phenolic Compounds in HHP-Treated Peels

The bioaccessibility of betalains and phenolic compounds in prickly pear peels is shown in Table 3. Graphical representation of data may be consulted in Supplementary Figure S2.

The bioaccessibility of indicaxanthin in untreated Sanguinos and Pelota peels was 62% and 55%, respectively. In Sanguinos peels, all pressurized samples had lower indicaxanthin bioaccessibility (−11% to −58%) than the untreated control. Meanwhile, Pelota peels treated at lower pressure-time combinations (100 MPa/CUT, 100 MPa/5 min and 350 MPa/CUT) showed no statistical differences (*p* ≤ 0.05) in indicaxanthin bioaccessibility compared to controls. Similarly, lower bioaccessibility values for betanin could be observed in peels treated with high hydrostatic pressure. The only increase (+27%) in betanin bioaccessibility was observed in Sanguinos peels treated at 600 MPa/5 min. In this study, HHP promoted the degradation of betalains, which had a negative effect on their bioaccessibility. It is likely that HHP enhanced the rupture of cell walls and favored enzymatic activity in the fruit tissues, which contributed to the loss of these compounds. Similarly, a previous study showed that betalain content decreased during HHP processing in beetroot juice [15]. It has been suggested that betalains are pressure-liable due to their high sensitivity to oxygen, pH, and enzymatic activity [16].

Submitting prickly pear peels to HHP caused little changes in the bioaccessibility of piscidic acid. The only significant increase (+32%) in the bioaccessibility of this phenolic acid was observed in Pelota peels treated at 600 MPa/5 min.

The bioaccessibility of 4-hydroxybenzoic acid glycoside was 61% and 66% in untreated Sanguinos and Pelota peels. Although 4-hydroxybenzoic acid in control samples had a high bioaccessibility, treating the samples with HHP resulted in an even higher bioaccessibility in the peels. The treatments with the highest bioaccessibility increase in 4-hydroxybenzoic acid glycoside were 600 MPa/CUT (+33% in Sanguinos and 35% in Pelota) and 100 MPa/5 min (+55% in Pelota). The rest of the assayed HPP conditions showed no statistically significant differences. Bioaccessibility increases of 4-hydroxybenzoic acid glycoside in pressurized peels are attributed to favorable changes in the food matrix by the HHP treatments (indirect effects) since little or no changes in phenolic acid content were observed post treatment.

Isorhamnetin glycoside bioaccessibility in prickly pear peels significantly improved with pressurization (Table 3). At the lowest and highest pressure-time combinations (100 MPa/CUT, 100 MPa/5 min and 600 MPa/5 min), the bioaccessibility of isorhamnetin glycosides was lower than in the correspondent controls. However, at intermediate-high pressure-time combinations, their bioaccessibility was similar (350 MPa/CUT) and even higher (350 MPa/5 min and 600 MPa/CUT). The bioaccessibility of isorhamnetin glucosyl-rhamnosyl-rhamnoside (IG1) in Pelota and Sanguinos prickly pears treated at 600 MPa/CUT was 120% and 84%, compared to 53% and 56% in untreated peels, respectively. Similarly, the bioaccessibility of isorhamnetin glucosyl-rhamnosyl-pentoside (IG2) was higher in Pelota (+36%) and Sanguinos (+40%) prickly pear peels processed at 600 MPa/CUT, compared to their controls. At this same condition, the bioaccessibility of isorhamnetin hexosyl-hexosyl-pentoside (IG3) was 63% and 94%, compared to 48% and 57% in untreated Pelota and Sanguinos peels, respectively.

Furthermore, the best treatments to improve the bioaccessibility of isorhamnetin glucosyl-pentoside (IG4) were at 350 MPa/CUT (+5 to 46% increase in bioaccessibility) and 600 MPa/CUT (+17 to 27% increases in bioaccessibility). Similarly, the best treatment to increase the bioaccessibility of isorhamnetin glucosyl-rhamnoside (IG5) in both Sanguinos and Pelota prickly pear peels was also at 600 MPa/CUT. Isorhamnetin glycoside extractability has been shown to be considerably enhanced in prickly pear peels submitted to high hydrostatic pressure, particularly at 350 MPa/CUT, 350 MPa/5 min, and 600 MPa/CUT [11]. This suggests that the high bioaccessibility of isorhamnetin glycosides in prickly pear peels could be driven by enhanced extractability due to HHP processing (direct effect). Previous studies have shown that high pressure increases the content of phenolic substances due to the breakdown of the cell wall structure and hydrolysis of polysaccharides [17]. This disruption of the plant cell walls induced by high hydrostatic pressure results in the release of bioactive compounds and mineral and starch content into the extracellular environment [18]. A previous study also showed the release of phenolic compounds from the cell walls and the modification and rearrangement of microfibrilated cellulose in prickly pear chlorenchyma cells treated with HHP [10].

The best HHP treatment to improve the bioaccessibility of phenolic bioactive compounds in prickly pear peels was 350 MPa/5 min because it increased the extractability of 4-hydroxybenzoic acid derivative and isorhamnetin glycosides. Hence, the digestive stability of bioactive compounds during each stage of digestion in prickly pear peels treated at 600 MPa/CUT is reported and discussed in Section 2.5.2.

### 2.5. Digestive Stability of Betalains and Phenolic Compounds

The best HHP treatments to achieve a higher bioaccessibility of bioactive compounds in prickly pear fruits were selected. For pulps, the highest bioaccessibility was obtained at 350 MPa/5 min. For peels, the highest bioaccessibility was obtained at 600 MPa/CUT. In this section, the concentration and the recovery of each bioactive compound in each phase of the simulated gastrointestinal digestion (oral, gastric, and intestinal phases) was studied. This information allowed us to elucidate potential mechanisms that could contribute to the effects of HHP treatments, and how they affect the bioaccessibility of betalains and phenolic compounds in pressurized prickly pear fruits.

#### 2.5.1. Stability and Recovery of Bioactives in Prickly Pear Pulps Treated with HHP at 350 MPa/5 min

The best treatment for increasing the bioaccessibility of most bioactive compounds in prickly pear pulps of both studied varieties was at 350 MPa/5 min. The content and recovery (%) of each bioactive compound in the pulp (control) and in oral, gastric, and intestinal phases of the simulated gastrointestinal digestion is shown in Table 4. The graphical representation of this data is available in the Supplementary Figure S3.

In pulps treated at 350 MPa/5 min, indicaxanthin showed a similar digestive stability to its respective controls (Table 4).

Pelota pulps treated at 350 MPa/5 min had a similar initial betanin content (25.8 mg/100 g fresh weight) to unpressurized pulps (27.9 mg/100 g fresh weight) [11]. However, this betacyanin showed a better digestive stability than indicaxanthin (Table 4). During gastro-intestinal digestion, pressurized pulps from both prickly pear varieties had statistically significant (*p* ≤ 0.05) higher betanin content in the intestinal phase, than their respective controls. The recoveries of Sanguinos and Pelota in the intestinal phase were 65% and 71%, respectively. These results support the theory that increases in betanin bioaccessibility are mainly driven by changes in the food matrix as an indirect effect of HHP. It has been suggested that processing technologies (such as HHP) influence enzymatic pectin conversion reactions [19,20]. This could have significant effects on pectin by denaturing of pectinases, enhancing the catalytic activity of pectinases and enhancing nonenzymatic (chemical) pectin conversions [21]. In the present study, gelatinization was observed visually after processing prickly pear pulps at 350 MPa/5 min. In more viscous pulps, betanin could be less exposed to enzymes and pH shifts in the gastrointestinal tract, which could have contributed to its higher digestive stability in pressurized samples than in unpressurized samples.

It has been shown that dominant factors involved in the influence of dietary fiber on digestion are (i) physical trapping of bioactive compounds within structured assemblies such as fruit tissue, and (ii) enhanced viscosity of gastric fluids restricting the peristaltic mixing process that promotes transport of enzymes to their substrates, bile salts to un-micellized fat, and soluble antioxidants to the gut wall [22]. Secondary factors may include binding of bile salts (and perhaps enzymes) to specific fiber components and inhibition of diffusion [23]. These effects of dietary fiber on digestion can be modified when applying HHP. In this study it is likely that treatments such as 350 MPa/5 min promoted changes in soluble fiber (i.e., pectins) from high methoxyl to low methoxyl. Low methoxyl pectins can increase the viscosity of the digesta and limit the interactions of betalains and digestive enzymes among other digestive components, which may degrade these antioxidants.

In the case of phenolic acids, piscidic acid in prickly pear pulps treated at 350 MPa/5 min (Table 4) showed both higher extractability and better digestive stability than unpressurized pulps. Higher digestive stability of piscidic acid in HHP-treated Pelota pulps is evidenced by the increasing content observed in each phase of the simulated gastrointestinal digestion; however, the pressurized Sanguinos pulp showed only a statistically significant (*p* ≤ 05) higher content in the gastric phase compared to its control. It is possible that HHP treatments could promote interactions between the prickly pear phenolic compounds and dietary fiber, due to the modifications in the tissue microstructure [10] producing the liberation of linked phenolics to the cell walls, which include the formation of junctions stabilized by an array of noncovalent bonds between hydroxide groups from phenolic compounds and polar groups from polysaccharide molecules (hydrogen bonds, electrostatic and dipolar interactions, and van der Waals attractions) [24]. Because these bonds are individually weak, their formation and disruption often occur as sharp, cooperative processes in response to comparatively small changes (pH or solvent quality in the gastrointestinal tract) [23].

In pulps processed at 350 MPa/5 min, differences in 4-hydroxybenzoic derivative content in the intestinal phase correlate with its initial content in the pressurized material. The stability of 4-hydroxybenzoic acid glycoside during the gastro-intestinal digestion was similar in treated and untreated prickly pear pulps. High recoveries in the gastric phase of all samples were observed (127% to 164%). However, the recovery of 4-hydroxybenzoic acid glycoside in the intestinal phases of Pelota and Sanguinos pulps treated with HHP was higher than in their untreated pulps.

#### 2.5.2. Stability and Recovery of Bioactives in Prickly Pear Peels Treated with HHP at 600 MPa/CUT

As shown previously, the pressurization of prickly pear peels at 600 MPa/CUT (come-up time) enhanced the bioaccessibility of 4-hydroxybenzoic acid glycoside (+33 to +37%) and isorhamnetin glycosides (+15 to +125%) in Sanguinos and Pelota varieties. This HHP treatment was chosen to study the digestive stability and recovery of its bioactive compounds because it was considered the best treatment to obtain the higher values of bioaccessibility for phenolic compounds (even when the betalain bioaccessibility decreased at this HHP treatment). Hence, the digestive stability and recovery of betalains, phenolic acids, and flavonoids in pulps treated at 600 MPa/CUT was studied in each stage of in vitro simulated gastrointestinal digestion to identify the factors that could influence their bioaccessibility (Table 5). The graphical representation of this data may be consulted in Supplementary Figure S4.

Betalain compounds, betanin and indicaxanthin, in prickly pear peels processed at 600 MPa/CUT suffered a considerable degradation due to the HPP treatment (−12 to −77%). Mentioned degradation of these pigments due to pressurization contributed to their lower content in the intestinal phase and bioaccessibility. A group of researchers applied HHP treatments of 650 MPa at different processing times (3, 7, 15, and 30) on beetroot slices (var. Red cloud) as an alternative to blanching pretreatment (90 °C for 7 min) and observed higher betalain degradation at higher time-exposure to high pressure [18].

The bioaccessibility (Table 3) and digestive stability (Table 5) of piscidic acid in prickly pear peels treated at 600 MPa/CUT showed no statistically significant differences compared to the control. On the other hand, 4-hydroxybenzoic acid glycoside showed noticeable increases in the gastric phase of HHP-treated Pelota prickly pear peels (Table 5). A previous study showed that treating prickly pear peels at 600 MPa/10 min at 22 °C and 55 °C increased the content of low molecular weight fractions of soluble dietary fiber [25]. An increase in low molecular weight soluble dietary fiber could have a positive effect on the digestive stability of phenolic acids in the gastric phase of digestion.

Regarding flavonoids, most isorhamnetin glycoside (IG) were more abundant in peels treated at 600 MPa/CUT than in their respective controls (Table 5). This contributed greatly to their high bioaccessibility. Pelota peels treated with HHP showed higher IG1 content throughout the complete digestive process. This included the intestinal phase where the recovery of HHP-treated Pelota peels was 62% compared to 53% in untreated ones. However, IG1 content in Sanguinos peels was not statistically different between pressurized and control samples. In other terms, the digestive stability of IG2 in HHP-treated prickly pear peels showed different behavior (Table 5). IG2 content in Pelota peels treated at 600 MPa/CUT was higher in the starting material (0.64 mg/100 g fresh weight), which contributed to its higher content in the intestinal phase (direct effect). However, in pressurized Sanguinos peels, IG2 content was similar in the starting material, but reached a higher concentration in the intestinal phase due to a higher digestive stability in the gastric phase (+107% higher than the control) (indirect effect).

HHP treatments did not enhance the release of this IG3 at 600 MPa/CUT (Table 5). Contrarily, in Sanguinos peels treated with HHP, IG4 showed no statistically significant differences in the intestinal phase, despite showing a high recovery of 120% in the gastric phase. Pressurized and control Pelota peels did not show any differences in IG4 content in the intestinal phase, either. Finally, a higher IG5 content was observed throughout the whole digestive process in Pelota peels. Despite higher content in the intestinal phase, Pelota showed lower (−23%) overall recovery compared to untreated peels.

## 3. Materials and Methods

### 3.1. Solvents, Reagents, and Standards

Ultra-pure water was obtained from a Milipak^®^ Express 40 system (Merck-Milipore, Dormstadt, Germany). Methanol (99.8% LC-MS) was purchased from VWR International (Barcelona, Spain). Pepsin (P6887; 791 U mg/L), α-amylase (10080; 79 U mg/L), pancreatin (P7545; 17 U/mg), bile (B8381), and other reagents used for the in vitro digestion assay were purchased from Sigma-Aldrich (St. Louis, MO, USA).

Piscidic acid was purified from prickly pear peels by semi-preparative high-performance liquid chromatography (HPLC) [12]. Betanin was purified from a betalain-rich concentrate extracted from commercial beetroot and indicaxanthin was semi-synthesized using purified betanin [12]. Commercial standards isorhamnetin and 4-hydroxybenzoic acid were purchased from Sigma-Aldrich (St. Louis, MO, USA). All isolated and semi-synthetized standards were analyzed for authenticity and purity by HPLC-ESI-MS-QTof.

### 3.2. Prickly Pear Fruits

**Figure 1:**
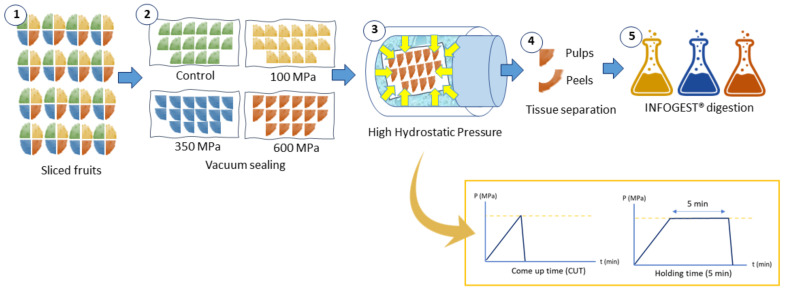
Separation scheme of prickly pear fruit sliced quarters to process with high hydrostatic pressure (HHP) at 100, 300, and 600 MPa and evaluate the effect of the come-up time (CUT) and holding time at each pressure.

Pelota prickly pears were provided by Agroproductores La Flor de Villanueva in San Sebastián Villanueva Acatzingo (Puebla, Mexico; 19°1’ N, 97°4′ W; 121 m. a. s. l.). Sanguinos prickly pears were purchased from Bioarchen in Archen (Murcia, Spain; 38°7′ N, 1°180′ W; 121 m. a. s. l.). Fruits were selected according to size, peel coloration, and ripeness, and were prepared for HHP treatments according to Figure 1. For each treatment, 16 fruits were sliced into quarters and one quarter of each fruit was placed in a bag (four bags of identical composition). Then, samples were vacuum sealed and treated with high hydrostatic pressure. Afterwards, samples were separated into pulps (mesocarp) and peels (endocarp and exocarp) and were frozen with liquid nitrogen. Samples were freeze dried at −45 °C and 1.3 × 10^−3^ MPa for 5 days (LyoBeta 15, Azbil Telstar, S.L., Terrasa, Spain). Freeze-dried prickly pear tissues were pulverized in a knife mill (Grindomix GM200, Retsch, Germany) to a small particle size (< 2 mm) and sieved to remove seeds in pulps. Samples were vacuum-sealed and stored at −20 °C until analysis.

### 3.3. High Hydrostatic Pressure Treatments

Prickly pears fruits were processed as described previously [11]. The purple Pelota variety was processed in Mexico in a pilot-scale high pressure equipment (Model 2 L, Flow Autoclave Systems, Columbus, OH, USA) and the red Sanguinos variety was processed in Spain in an equipment of comparable characteristics (Model 2 L, Stansted SFP 7100:9/2C, Harlow, Essex UK). Pressure conditions were based on commercially used intensities of 100 MPa (low), 350 MPa (intermediate), and 600 MPa (high) and time (5 min). The effect of heir come-up times (CUTs) was evaluated as well. Compression rates were 7 MPa/s and decompression occurred in under 1 s. The come-up times (CUTs) at 100, 350, and 600 MPa were 14.3 ± 1, 50.0 ± 4, and 85.7 ± 6 s, respectively. Pressure, time, and temperature were controlled by a computer program. Average maximum temperatures reached in the Flow Autoclave Systems (Pelota variety) were 17, 25, and 32 °C for CUT and 19, 27, and 34 °C for holding times at 100, 350, and 600 MPa, respectively. In both varieties, the average maximum temperature reached was 20 °C since it was constantly cooled by means of a thermostat jacket. The processing of each treatment was performed three times.

### 3.4. In Vitro Simulated Gastrointestinal Digestion

The in vitro simulated gastrointestinal digestion was performed according to the standardized INFOGEST protocol [7,26] using prickly pear rehydrated puree (control and HPP treated samples). Prior to digestion, the rehydrated puree was extracted according to [12] to analyze the concentration of bioactive compounds in the starting material. The simulated saliva fluid, simulated gastric fluid, and simulated duodenal fluid were prepared according to [7]. The oral phase was made up of 5 g rehydrated fruit puree, 4 mL of electrolyte stock solution (SSF), 0.025 mL of 0.3 M CaCl_2_(H_2_O)_2_, 0.75 mL salivary amylase (75 U/mL activity; 10 mg/mL concentration), and 0.225 mL of water. The gastric phase was made up of 10 mL from the oral phase, 8 mL of electrolyte stock solution (SGF), 0.005 mL of 0.3 M CaCl_2_(H_2_O)_2_, 0.667 mL of pepsin (2000 U/mL activity, 20 mg/mL concentration), 0.928 mL of water, and approximately 0.4 mL of HCL (5 M) to adjust the pH to 3. The intestinal phase was composed of 20 mL from the gastric phase, 8 mL of electrolyte stock solution, 0.04 mL of CaCl_2_(H_2_O)_2_, 5 mL of trypsin in pancreatin (100 u/mL activity, 133.3 mg/mL concentration), 3 mL of bile acid mixture from bovine and ovine (10 mM), 3.16 mL of water, and approximately 0.8 mL of NaOH (5 M) to adjust the pH to 7. Enzymes were prepared and added to the simulated fluids prior to the digestive assay.

To study bioaccessibility, all untreated and HHP-treated prickly pears were submitted to the complete digestive simulation and their intestinal phases were frozen with liquid nitrogen and stored at −20 °C. Samples were extracted from thawed intestinal phases and analyzed by HPLC-DAD-MS.

The bioaccessibility of betalains and phenolic compounds was calculated according to Equation (1). This quantification differs from the traditional computation which, by definition, divides the bioactive content in HHP-treated fruit by the bioactive content in the intestinal phase of HHP-treated fruit. However, when talking about processed foods, any positive or negative effects on bioactive compounds due to processing would directly influence the bioaccessibility parameter, providing false positive or false negative effects. Therefore, to assess the bioaccessibility of all HHP-treated samples in a first instance, the bioactive content in the untreated fruit was divided by the bioactive content in the intestinal phase of the HHP-treated fruit tissue.

(1) $$Bioaccessibility\;\left(\%\right)=\frac{Bioactive\;content_{untreated\;fruit}}{Bioactive\;content_{intestinal\;phase\;of\;HHP\;treated\;fruit}}$$

After analyzing this bioaccessibility data of *Opuntia* samples, we conducted the study of the digestive stability of selected samples chosen on the basis on the HHP treatments, which resulted in the highest bioaccessibility. These selected HPP-treated samples were digested again, and a collection of each digestive phase (oral, gastric and intestinal) fractions were made in order to analyze the bioactive compounds by HPLC-DAD-MS. These digesta were frozen immediately with liquid nitrogen and stored at −20 °C. Samples were extracted from thawed digestive phases and analyzed by HPLC-DAD-MS.

The stability of betalains and phenolic compounds in each digestive phase (oral, gastric and intestinal) of simulated gastrointestinal digestion is shown in Equation (2). This stability could be expressed by the recovery of bioactive compounds during simulated gastrointestinal digestion and provides relevant information related to digestive stability and was calculated by dividing the bioactive content in samples treated with HHP by the bioactive content in the digestive phase of HHP-treated samples.

(2) $$Recovery\;\left(\%\right)=\frac{Bioactive\;content_{HHP\;treated\;fruit}}{Bioactive\;content_{digestive\;phase\;of\;HHP\;treated\;fruit}}$$

### 3.5. Extraction of Betalains and Phenolic Compounds for HPLC Analysis

Betalains and phenolic compounds were extracted simultaneously from fruit tissues [12]. One gram of freeze-dried sample was extracted with 5 mL of methanol:water in a 1:1 (*v:v*) proportion. Samples were vortexed and sonicated for 4 min. Then they were centrifuged at 4 °C at 10,000× *g* for 10 min. The supernatant was recovered and the pellet was re-extracted two more times with methanol:water and one last time with pure methanol, while recovering the supernatant after each extraction. The supernatants were combined and concentrated in a rotavapor by evaporating methanol. The aqueous extracts were then made up to 5 mL with water, filtered, and analyzed by HPLC.

Betalains and phenolic compounds were extracted simultaneously from thawed digestive phases according to the procedure described by [27]. An aliquot of the digestive phase was weighed (10g oral phase or 20 g gastric and intestinal phase) and placed in an assay tube. The pH was adjusted to 4 and pure methanol was added in a 1:1 (*v:v*) proportion. The sample was homogenized for 2 min at 700× *g* using a ultrahomogenizer (Omnimixer ES-207, Omni International Inc, Gainsville, FL, USA) in an ice bath and then centrifuged at 4 °C for 15 min at 9000 rpm. The supernatant was concentrated using a rotavapor and the sample was made up to 5 mL (oral phase) or 20 mL (gastric and intestinal phase), then filtered for HPLC analysis.

### 3.6. Quantification of Betalains and Phenolic Compounds by HPLC

Betalains and phenolic compounds were quantified simultaneously by high-performance liquid chromatography using a 1200 Series Agilent HPLC System (Agilent Technologies, Santa Clara, CA, USA) with a reverse-phase C18 column (Zorbax SB-C18, 250 × 4.6 mm i.d., S-5 µM; Aglient) at 25 °C [10,12]. Mobile phase A was 1% formic acid (*v/v*) in ultrapure water and mobile phase B was 1% formic acid (*v/v*) in methanol. Separation was achieved using an initial composition of 15% B during 15 min, increased to 25% within 10 min, subsequentially ramped to 50% B within 10 min, increased to 75% B in 15 min, and finally followed by a decrease period of 15% B in 5 min prior to isocratic re-equilibration for 10 min. The flow rate was 0.8 mL/min and the injection volume was 20 μL. The UV-vis photodiode array detector was set at four wavelengths to detect phenolic acids (280 nm), flavonoids (370 nm), betaxanthins (480 nm), and betacyanins (535 nm). UV/Vis spectra were also recorded between 200 and 700 nm. The HPLC-DAD was coupled to a mass spectrometry detector (LCMS SQ 6120, Agilent, Agilent Technologies, Santa Clara, CA, USA) with an electrospray ionization (ESI) source operating in positive ion mode. The drying gas was nitrogen at 3 L/ min at 137.9 KPa. The nebulizer temperature was 300 °C and the capillary had 3500 V potential. The coliseum gas was helium, and the fragmentation amplitude were 70 V. Spectra were recorded m/z from 100 to 1000.

Further mass spectrometry analyses were performed in a maXis II LC-QTOF equipment (Bruker Daltonics, Bremen, Germany) with an ESI source and the same chromatographic conditions. The ESI-QTOF detector worked in positive ion mode and recorded spectra *m/z* from 50 to 3000. Operation gas at 6 L/min. MS/MS analysis used the bbCID (Broad Band Collision Induces Dissociation) method at 30 eV.

Compounds were identified and quantified according to their retention times, UV/Vis and mass spectral data compared to their respective standards and calibration curves. These are in agreement with what was reported previously by various authors [12,28,29,30]. For indicaxanthin standard, the retention time was 10.5 min; UV λ_max_ at 478 nm; [M + H]^+^ ion at 309.11 *m/s*; MS/MS fragments at *m/s* 263.10, 217.10, 70.06; and calibration curve was y = 0.0105x (R^2^ = 0.9974) where x = peak area and y = concentration (ug/mL). For betanin standard, the retention time was 15.7 min; UV λ_max_ at 534 nm; [M + H]^+^ ion at 551.15 *m/s*; MS/MS fragments at *m/s* 390.10, 389.10; and calibration curve was y = 0.0175x (R^2^ = 0.9944), where x = peak area and y = concentration (ug/mL). For hydroxybenzoic acid standard, the retention time was 23.5 min; UV λ_max_ at 274 nm; [M+H]^+^ ion at 205.05; and calibration curve was y = 0.0409x (R^2^ = 0.9999) where x = peak area and y = concentration (ug/mL). For piscidic acid standard, the retention time was 14.0 min; UV λ_max_ at 232 and 275 nm; [M + H]^+^ ion at 257.07 *m/s*; MS/MS fragments at *m/s* 191.07, 147.04, and 119.05; 107.05; and calibration curve was y = 0.4341x (R^2^ = 0.9967) where x = peak area and y= concentration (ug/mL). Isorhamnetin glycosides (IG1, Rt = 40.0 min; IG2, Rt = 40.4 min; IG3, Rt = 40.9 min; IG4, Rt = 41.2 min; and IG5, Rt = 44.5 min) all showed the MS/MS fragment at *m/s* 317.07 for isorhamnetin. Hence, they were quantified with the isorhamnetin standard. For isorhamnetin standard, the retention time was 49.9 min; UV λ_max_ at 370 nm; [M + H]^+^ ion at 317.07 *m/s*; and calibration curve was y = 0.0324x (R^2^ = 0.9820) where x = peak area and y= concentration (ug/mL). Details on the elucidation of the isorhamnetin glycosides were reported in previous research articles [5,12,31].

### 3.7. Statistical Analysis

Data were expressed as mean ± standard deviation of three determinations. Significant differences were calculated by one-way analysis of variance (ANOVA) and a post hoc Tukey’s test (*p* ≤ 0.05). Pearson’s correlation was calculated at *p* ≤ 0.05 and *p* ≤ 0.01. Statistical analyses were performed with IBM SPPS Statistics 23.0 (IBM Corp, Armonk, NY, USA).

## 4. Conclusions

Sanguinos and Pelota prickly pear fruits were treated with high hydrostatic pressure (100, 350, and 600 MPa; CUT and 5 min), which had different effects on the bioaccessibility of their betalains, phenolic acids, and flavonoids in their peel and pulp tissue-sections. The bioaccessibility of betalains in pressurized prickly pear tissue-sections was mostly negatively affected by their degradation during processing. However, the bioaccessibility of betalains in pulps could be enhanced by HHP at specific conditions such as 350 MPa/5min. This effect was a result of a higher digestive stability in the gastric and intestinal phase of simulated digestion, which is likely influenced by HPP-induced changes to soluble dietary fiber. Phenolic acids were more pressure-resistant than betalains and their bioaccessibility was mostly favored by changes to the food matrix, which enhanced their extractability in the gastric phase of digestion. Curiously, the effect of HHP on isorhamnetin glycosides (flavonoids) depended greatly on the type of glycoside and pressurization conditions. The bioaccessibility of isorhamnetin glycosides in peels was greatly favored at 600 MPa/CUT by both effects of (i) enhanced extractability and (i) higher digestive stability.

In general terms, the best treatments for enhancing the bioaccessibility of bioactive compounds in prickly pear pulps (edible fraction) was at 350 MPa/5 min, and in peels (potential healthy ingredient) was at 600 MPa/CUT. Future studies require the assessment of changes in polysaccharides such as dietary fiber and, more specifically, pectic substances to study the interactions between bioactive compounds and the food matrix. In this study, we expect to contribute to the use of high hydrostatic pressure to promote the healthy attributes of foods by increasing the bioaccessibility of their bioactive compounds. To take advantage of the nutritional aspects presented in this work, HPP-treated prickly pear fruits can be further processed into juices, purees, and jams. Further research regarding the shelf life stability of HHP-treated prickly pear fruit products to promote their commercialization is needed.

## Figures and Tables

**Table 1:** Betalain and phenolic content (mg/100 g fresh weight) in rehydrated Sanguinos and Pelota prickly pear (*Opuntia ficus-indica* L. Mill.) peels and pulps.

	Sanguinos		Pelota	
Compound	Pulp	Peel	Pulp	Peel
Indicaxanthin	0.90 ± 0.05	0.60 ± 0.01	3.92 ± 0.24 *	1.01 ± 0.01 *
Betanin	2.05 ± 0.02	5.78 ± 0.23	27.91 ± 0.45 *	16.29 ± 0.10 *
Piscidic acid	58.43 ± 3.53	439.45 ± 0.90	195.63 ± 1.48 *	779.17 ± 4.74 *
4-hydroxybenzoic acid glycoside	1.25 ± 0.13	19.12 ± 0.21	4.47 ± 1.17	23.62 ± 0.14 *
Isorhamnetin glucosyl-rhamnosyl-rhamnoside (IG1)	n.d.	0.55 ± 0.03	n.d.	1.44 ± 0.01 *
Isorhamnetin glucosyl-rhamnosyl-pentoside (IG2)	n.d.	1.51 ± 0.13	n.d.	0.35 ± 0.00 *
Isorhamnetin hexosyl-hexosyl-pentoside (IG3)	n.d.	0.32 ± 0.03	n.d.	0.15 ± 0.00 *
Isorhamnetin glucosyl-pentoside (IG4)	n.d.	0.81 ± 0.06	n.d.	0.23 ± 0.00 *
Isorhamnetin glucosyl-rhamnoside (IG5)	n.d.	3.09 ± 0.18	n.d.	2.26 ± 0.01 *

Results are expressed as mean ± standard deviation (*n* = 3). Lowercase superscript letters indicate statistically significant differences (*p* ≤ 0.05) between peels and pulps. * Indicate statistically significant differences (*p* ≤ 0.05) between varieties. n.d. not detected.

**Table 2:** Initial content (mg/100 g weight) and in vitro bioaccessibility of betalains and phenolic compounds in HHP-treated (100 MPa, 350 MPa, 600 MPa; CUT and 5 min) Sanguinos and Pelota prickly pear pulps and in untreated (control) pulps.

		Prickly Pear (*Opuntia ficus-indica*) Pulps			
		Sanguinos		Pelota	
	Treatment	Pulp (mg/100 g Weight) ^1^	Bioaccessibility (%)	Pulp (mg/100 g Weight) ^1^	Bioaccessibility (%)
Indicaxanthin	Control	0.90 ± 0.05^a^	53.05 ± 3.01^c^	3.92 ± 0.24^c^	54.87 ± 2.49^bc^
	100 MPa/CUT	0.73 ± 0.05^a^	40.93 ± 3.27^b^	3.62 ± 0.17^bc^	52.81 ± 5.17^bc^
	350 MPa/CUT	0.90 ± 0.07^a^	44.81 ± 2.24^bc^*	5.38 ± 0.05^d^	64.83 ± 5.19^c^
	600 MPa/CUT	0.72 ± 0.06^a^	25.30 ± 2.02^a^*	2.85 ± 0.16^ab^	36.78 ± 2.94^a^
	100 MPa/5 min	0.86 ± 0.07^a^	28.84 ± 2.31^a^*	3.51 ± 0.14^bc^	46.87 ± 3.28^ab^
	350 MPa/5 min	0.84 ± 0.06^a^	46.98 ± 3.29^bc^	3.21 ± 0.08^abc^	55.38 ± 2.22^bc^
	600 MPa/5 min	0.71 ± 0.01^a^	22.33 ± 1.79^a^*	2.65 ± 0.50^a^	43.12 ± 3.45^ab^
Betanin	Control	2.05 ± 0.02^a^	42.49 ± 2.69^c^	27.91 ± 0.45^ab^	44.92 ± 1.52^ab^
	100 MPa/CUT	1.80 ± 0.07^a^	35.10 ± 2.81^bc^	28.44 ± 0.73^ab^	48.50 ± 4.74^b^
	350 MPa/CUT	2.32 ± 0.23^a^	43.01 ± 3.44^c^	35.02 ± 0.00^b^	50.65 ± 4.05^b^
	600 MPa/CUT	2.14 ± 0.22^a^	28.67 ± 1.43^ab^*	27.93 ± 1.64^ab^	39.23 ± 3.14^ab^
	100 MPa/5 min	1.98 ± 0.14^a^	25.98 ± 1.82^ab^*	26.44 ± 1.79^a^	35.21 ± 2.46^a^
	350 MPa/5 min	2.19 ± 0.23^a^	69.57 ± 5.57^d^*	25.79 ± 0.27^a^	65.88 ± 2.64^c^
	600 MPa/5 min	2.02 ± 0.23^a^	20.51 ± 1.64^a^*	24.79 ± 4.91^a^	46.95 ± 3.76^ab^
Piscidic acid	Control	58.43 ± 3.53^a^	53.61 ± 4.29^ab^*	195.63 ± 1.48^a^	38.49 ± 3.08^a^
	100 MPa/CUT	63.32 ± 7.10^a^	56.66 ± 4.53^abc^*	255.44 ± 15.40^bc^	82.25 ± 6.58^cd^
	350 MPa/CUT	71.46 ± 1.26^ab^	58.61 ± 3.44^abc^	223.96 ± 3.82^ab^	62.71 ± 5.02^bc^
	600 MPa/CUT	92.66 ± 9.61^b^	65.68 ± 5.25^bc^	311.03 ± 17.41^d^	71.56 ± 3.58^bcd^
	100 MPa/5 min	62.78 ± 1.89^a^	73.70 ± 1.05^cd^*	235.34 ± 15.86^abc^	54.39 ± 3.81^ab^
	350 MPa/5 min	73.82 ± 8.70^ab^	89.36 ± 7.15^d^	273.68 ± 2.09^bc^	106.20 ± 8.50^e^
	600 MPa/5 min	81.97 ± 9.50^ab^	42.40 ± 3.39^a^*	287.35 ± 29.07^cd^	88.43 ± 7.07^de^
4-hydrozybenzoic acid glycoside	Control	1.25 ± 0.13^a^	20.00 ± 1.60^a^	4.47 ± 1.17^a^	17.00 ± 1.36^a^
	100 MPa/CUT	1.54 ± 0.40^ab^	28.53 ± 2.79^b^	5.76 ± 0.45^ab^	25.89 ± 2.07^b^
	350 MPa/CUT	1.87 ± 0.17^abc^	27.43 ± 2.19^ab^	4.09 ± 0.03^a^	21.09 ± 1.24^ab^
	600 MPa/CUT	2.48 ± 0.28^c^	20.28 ± 1.62^a^	9.87 ± 0.47^c^	16.76 ± 1.34^a^
	100 MPa/5 min	1.60 ± 0.07^ab^	22.10 ± 1.55^ab^	7.69 ± 0.84^bc^	22.81 ± 1.82^ab^
	350 MPa/5 min	2.18 ± 0.07^bc^	47.13 ± 1.89^c^	6.70 ± 0.14^ab^	38.44 ± 3.08^c^
	600 MPa/5 min	2.19 ± 0.27^bc^	19.18 ± 1.53^a^*	8.38 ± 1.69^bc^	37.48 ± 3.00^c^

Results are expressed as mean ± standard deviation (*n* = 3). Superscript letters indicate statistically significant differences (*p* ≤ 0.05) between treatments. * Indicates statistically significant differences (*p* ≤ 0.05) in bioaccessibility between Pelota and Sanguinos varieties. ^1^ Data previously reported [11].

**Table 3:** Initial content (mg/100 g weight) and in vitro bioaccessibility of betalains and phenolic compounds in HHP-treated (100 MPa, 350 MPa, 600 MPa; CUT and 5 min) Sanguinos and Pelota prickly pear peels and in untreated (control) peels.

		Prickly Pear (*Opuntia ficus-indica*) Peels			
		Sanguinos		Pelota	
Compound	Treatment	Peel (mg/100 g Fresh Weight) ^1^	Bioaccessibility (%)	Peel (mg/100 g Fresh Weight) ^1^	Bioaccessibility (%)
Indicaxanthin	Control	0.60 ± 0.01^b^	61.98 ± 3.70^d^	1.01 ± 0.01^c^	55.39 ± 2.90^d^
	100 MPa/CUT	0.53 ± 0.01^ab^	23.64 ± 1.89^ab^*	0.85 ± 0.06^bc^	49.35 ± 3.95^cd^
	350 MPa/CUT	0.58 ± 0.03^ab^	41.54 ± 3.32^c^	0.80 ± 0.01^b^	48.92 ± 3.91^cd^
	600 MPa/CUT	0.53 ± 0.03^ab^	33.50 ± 4.55^bc^	0.57 ± 0.06^a^	38.58 ± 3.09^abc^
	100 MPa/5 min	0.55 ± 0.03^ab^	19.93 ± 1.59^a^*	0.74 ± 0.01^ab^	45.76 ± 3.66^bcd^
	350 MPa/5 min	0.62 ± 0.04^b^	37.82 ± 3.03^c^	0.92 ± 0.12^bc^	35.23 ± 2.82^ab^
	600 MPa/5 min	0.49 ± 0.00^a^	33.50 ± 4.21^bc^	0.55 ± 0.02^a^	30.24 ± 2.42^a^
Betanin	Control	5.78 ± 0.23^d^	28.88 ± 2.31^d^*	16.29 ± 0.10^d^	46.11 ± 3.69^b^
	100 MPa/CUT	3.77 ± 0.23^b^	3.58 ± 0.29^a^*	15.39 ± 0.89^cd^	29.54 ± 2.36^a^
	350 MPa/CUT	4.48 ± 0.38^bc^	11.98 ± 0.96^b^*	12.30 ± 0.00^b^	32.13 ± 2.57^a^
	600 MPa/CUT	4.52 ± 0.28^bc^	22.84 ± 1.83^c^*	13.92 ± 1.06^bc^	31.36 ± 2.51^a^
	100 MPa/5 min	3.76 ± 0.44^b^	1.17 ± 0.09^a^*	12.66 ± 0.24^b^	31.60 ± 2.53^a^
	350 MPa/5 min	5.26 ± 0.53^cd^	20.16 ± 1.61^c^*	15.39 ± 0.45^cd^	30.36 ± 2.43^a^
	600 MPa/5 min	2.02 ± 0.23^a^	36.80 ± 2.94^e^	10.30 ± 058^a^	32.36 ± 2.59^a^
Piscidic acid	Control	439.45 ± 0.90^c^	52.36 ± 4.19^a^	779.17 ± 4.74^ab^	52.71 ± 4.22^ab^
	100 MPa/CUT	358.85 ± 5.05^ab^	55.90 ± 4.47^a^	821.22 ± 60.40^ab^	53.79 ± 4.30^ab^
	350 MPa/CUT	398.36 ± 14.95^bc^	45.09 ± 3.61^a^	721.33 ± 0.18^ab^	59.40 ± 4.75^ab^
	600 MPa/CUT	346.51 ± 18.65^ab^	54.78 ± 4.38^a^	713.46 ± 59.11^ab^	46.83 ± 3.75^a^
	100 MPa/5 min	393.96 ± 13.37^bc^	48.68 ± 3.89^a^	689.55 ± 0.20^a^	55.06 ± 4.40^ab^
	350 MPa/5 min	385.99 ± 35.08^bc^	45.88 ± 3.67^a^	847.09 ± 31.12^b^	62.40 ± 4.99^ab^
	600 MPa/5 min	311.32 ± 0.51^a^	47.26 ± 3.78^a^*	750.60 ± 33.40^ab^	69.96 ± 5.60^b^
4-hydroxybenzoic acid glycoside	Control	19.12 ± 0.21^d^	60.67 ± 4.85^ab^	23.62 ± 0.14^a^	65.55 ± 5.24^a^
	100 MPa/CUT	16.37 ± 0.28^bc^	65.38 ± 5.23^abc^	24.12 ± 2.67^a^	63.29 ± 5.06^a^
	350 MPa/CUT	17.47 ± 1.61^cd^	50.81 ± 4.06^a^	21.04 ± 0.09^a^	67.86 ± 5.43^ab^
	600 MPa/CUT	14.38 ± 0.73^b^	80.54 ± 6.44^c^	25.67 ± 2.20^a^	89.34 ± 7.15^bc^
	100 MPa/5 min	19.54 ± 0.18^d^	74.89 ± 5.99^bc^	36.83 ± 1.04^b^	102.28 ± 8.18^c^
	350 MPa/5 min	19.08 ± 0.18^d^	61.62 ± 4.93^ab^	23.02 ± 0.90^a^	71.36 ± 5.71^ab^
	600 MPa/5 min	12.07 ± 0.02^a^	60.19 ± 4.82^ab^	25.92 ± 0.59^a^	80.91 ± 6.47^abc^
IG1 ^2^	Control	0.54 ± 0.02^ab^	56.20 ± 4.50^ab^	1.44 ± 0.01^b^	53.30 ± 4.26^bc^
	100 MPa/CUT	0.61 ± 0.03^bc^	82.13 ± 6.57^c^*	0.86 ± 0.08^a^	25.64 ± 2.05^a^
	350 MPa/CUT	0.63 ± 0.01^bc^	72.46 ± 5.80^bc^	2.39 ± 0.04^c^	76.75 ± 6.30^d^
	600 MPa/CUT	0.66 ± 0.06^c^	84.06 ± 6.72^c^*	2.79 ± 0.17^d^	120.15 ± 9.61^e^
	100 MPa/5 min	0.67 ± 0.04^c^	67.63 ± 5.41^abc^*	1.44 ± 0.05^b^	40.29 ± 3.22^ab^
	350 MPa/5 min	0.67 ± 0.01^c^	77.29 ± 6.18^c^*	3.21 ± 0.04^e^	108.06 ± 8.64^e^
	600 MPa/5 min	0.50 ± 0.00^a^	48.31 ± 3.86^a^	3.18 ± 0.01^e^	65.93 ± 5.27^cd^
IG2 ^3^	Control	1.50 ± 0.13^a^	41.80 ± 3.34^a^*	0.35 ± 0.00^b^	72.56 ± 5.80^bc^
	100 MPa/CUT	1.37 ± 0.24^a^	54.32 ± 4.35^bc^*	0.20 ± 0.01^a^	24.27 ± 1.94^a^
	350 MPa/CUT	1.44 ± 0.11^a^	40.56 ± 3.24^a^*	0.56 ± 0.03^c^	85.27 ± 6.82^cd^
	600 MPa/CUT	1.50 ± 0.06^a^	59.10 ± 4.73^c^*	0.64 ± 0.05^c^	99.02 ± 7.92^de^
	100 MPa/5 min	1.71 ± 0.12^a^	38.03 ± 3.04^a^	0.34 ± 0.01^b^	34.93 ± 2.79^a^
	350 MPa/5 min	1.70 ± 0.01^a^	43.88 ± 3.51^ab^*	0.73 ± 0.00^d^	110.44 ± 8.84^e^
	600 MPa/5 min	1.30 ± 0.00^a^	36.55 ± 2.92^a^*	0.74 ± 0.03^d^	63.44 ± 5.08^b^
IG3 ^4^	Control	0.32 ± 0.03^ab^	48.05 ± 3.84^c^	0.15 ± 0.00^c^	56.89 ± 4.55^bc^
	100 MPa/CUT	0.29 ± 0.03^ab^	42.56 ± 3.40^bc^*	0.11 ± 0.00^b^	23.52 ± 1.88^a^
	350 MPa/CUT	0.31 ± 0.02^ab^	33.20 ± 2.66^b^*	0.20 ± 0.01^d^	59.04 ± 4.72^bc^
	600 MPa/CUT	0.32 ± 0.01^ab^	62.51 ± 5.00^d^*	0.23 ± 0.01^e^	93.69 ± 7.50^d^
	100 MPa/5 min	0.36 ± 0.00^b^	31.78 ± 2.54^b^*	0.15 ± 0.00^c^	15.68 ± 1.25^a^
	350 MPa/5 min	0.34 ± 0.01^ab^	37.74 ± 3.02^bc^*	0.08 ± 0.00^a^	61.50 ± 4.92^c^
	600 MPa/5 min	0.28 ± 0.00^a^	16.63 ± 1.33^a^*	0.08 ± 0.02^a^	43.97 ± 3.52^b^
IG4 ^5^	Control	0.82 ± 0.06^ab^	59.49 ± 4.76^cd^	0.23 ± 0.00^b^	58.74 ± 4.70^bc^
	100 MPa/CUT	0.52 ± 0.20^a^	52.26 ± 4.18^c^*	0.12 ± 0.00^a^	22.68 ± 1.81^a^
	350 MPa/CUT	0.55 ± 0.09^ab^	61.83 ± 4.95^cd^	0.42 ± 0.00^c^	85.85 ± 6.87^de^
	600 MPa/CUT	0.71 ± 0.03^ab^	68.59 ± 5.49^d^	0.44 ± 0.04^cd^	75.20 ± 6.02^cd^
	100 MPa/5 min	0.87 ± 0.05^b^	21.56 ± 1.72^a^	0.23 ± 0.01^b^	24.88 ± 1.99^a^
	350 MPa/5 min	0.86 ± 0.01^b^	39.20 ± 3.14^b^*	0.49 ± 0.00^d^	99.19 ± 7.93^e^
	600 MPa/5 min	0.56 ± 0.01^ab^	35.00 ± 2.80^b^*	0.51 ± 0.04^d^	53.90 ± 4.31^b^
IG5 ^6^	Control	3.10 ± 0.18^b^	29.77 ± 2.38^b^*	2.27 ± 0.01^b^	55.45 ± 4.44^b^
	100 MPa/CUT	2.44 ± 0.18^ab^	43.05 ± 3.44^c^*	1.22 ± 0.06^a^	24.45 ± 1.96^a^
	350 MPa/CUT	2.12 ± 0.15^a^	26.81 ± 2.14^b^*	4.31 ± 0.45^c^	99.96 ± 8.00^c^
	600 MPa/CUT	2.82 ± 0.10^ab^	42.10 ± 3.37^c^*	5.10 ± 0.49^c^	97.67 ± 7.81^c^
	100 MPa/5 min	3.11 ± 0.35^b^	11.94 ± 0.96^a^*	2.20 ± 0.03^b^	39.91 ± 3.19^ab^
	350 MPa/5 min	3.14 ± 0.39^b^	28.05 ± 2.24^b^*	5.21 ± 0.05^c^	137.52 ± 11.00^d^
	600 MPa/5 min	2.16 ± 0.00^a^	25.38 ± 2.03^b^*	5.12 ± 0.45^c^	59.11 ± 4.73^b^

Results are expressed as mean ± standard deviation (*n* = 3). Superscript letters indicate statistically significant differences (*p* ≤ 0.05) between treatments. * Indicates statistically significant differences (*p* ≤ 0.05) in bioaccessibility between Pelota and Sanguinos varieties. ^1^ Data previously reported [11], ^2^ isorhamnetin glucosyl-rhamnosyl-rhamnoside (IG1), ^3^ isorhamnetin glucosyl-rhamnosyl-pentoside (IG2), ^4^ isorhamnetin hexosyl-hexosyl-pentoside (IG3), ^5^ isorhamnetin glucosyl-pentoside (IG4), ^6^ isorhamnetin glucosyl-rhamnoside (IG5).

**Table 4:** In vitro digestive stability (mg/100 g fresh weight) of betalains and phenolic compounds in HHP-treated (350 MPa/5 min) Sanguinos and Pelota prickly pear pulps.

	Treatment	Pulp	Oral Phase		Gastric Phase		Intestinal Phase	
		Content ^1^	Content ^1^	Recovery (%)	Content ^1^	Recovery (%)	Content ^1^	Recovery (%)
Indicaxanthin	Sanguinos Control	0.90 ± 0.05^c^	0.82 ± 0.02^bc^	91 ± 3^b^	0.75 ± 0.02^b^	83 ± 3^b^	0.48 ± 0.03^a^	53 ± 3^a^
	Sanguinos 350 MPa/5 min	0.84 ± 0.06^a^	1.10 ± 0.06^a^	131 ± 16^b^	1.01 ± 0.34^a^	120 ± 11^b^	0.42 ± 0.03^a^	51 ± 4^a^
	Pelota Control	3.92 ± 0.24^b^	4.09 ± 0.32^b^	104 ± 2^c^	3.57 ± 0.09^b^	91 ± 3^b^	2.15 ± 0.17^a^	54 ± 2^a^
	Pelota 350 MPa/5 min	3.21 ± 0.08^b^	3.60 ± 0.43^b^	112 ± 11^b^	3.46 ± 0.17^b^*	108 ± 3^b^*	2.17 ± 0.17^a^	68 ± 5^a^
Betanin	Sanguinos Control	2.05 ± 0.02^d^	1.31 ± 0.07^c^	64 ± 3^c^	1.04 ± 0.05^b^	51 ± 2^b^	0.87 ± 0.06^a^	42 ± 3^a^
	Sanguinos 350 MPa/5 min	2.19 ± 0.23^ab^	3.67 ± 0.08^b^*	168 ± 14^b^*	3.24 ± 0.79^b^	148 ± 21^b^*	1.42 ± 0.11^a^*	65 ± 5^a^*
	Pelota Control	27.91 ± 0.45^c^	29.68 ± 1.31^c^	106 ± 3^c^	25.76 ± 0.82^b^	92 ± 1^b^	12.54 ± 0.50^a^	45 ± 3^a^
	Pelota 350 MPa/5 min	25.79 ± 0.27^b^*	23.54 ± 2.01^b^	91 ± 7^a^	22.78 ± 1.47^b^	88 ± 5^a^	18.39 ± 1.47^a^*	71 ± 6^a^*
Piscidic acid	Sanguinos Control	58.43 ± 3.53^b^	50.40 ± 2.52^b^	86 ± 1^b^	54.87 ± 2.74^b^	94 ± 1^c^	31.32 ± 2.51^a^	54 ± 4^a^
	Sanguinos 350 MPa/5 min	73.82 ± 8.70^ab^	78.30 ± 12.98^ab^	106 ± 5^b^*	107.36 ± 17.19^b^*	145 ± 6^c^*	52.21 ± 4.18^a^*	71 ± 6^a^*
	Pelota Control	195.63 ± 1.48^d^	163.24 ± 1.52^c^	83 ± 0^c^	106.36 ± 3.62^b^	54 ± 1^b^	75.30 ± 6.02^a^	38 ± 3^a^
	Pelota 350 MPa/5 min	273.68 ± 2.09^b^*	267.30 ± 25.06^b^*	98 ± 8^a^	238.01 ± 19.18^ab^*	87 ± 6^a^	207.76 ± 16.62^a^*	76 ± 6^a^*
4-hydroxy benzoic acid derivative	Sanguinos Control	1.25 ± 0.13^b^	1.05 ± 0.05^b^	84 ± 5^b^	2.05 ± 0.10^c^	164 ± 9^c^	0.25 ± 0.02^a^	20 ± 2^a^
	Sanguinos 350 MPa/5 min	2.18 ± 0.07^b^*	3.06 ± 0.61^b^*	140 ± 23^b^	3.49 ± 0.50^b^	160 ± 18^b^	0.59 ± 0.05^a^*	27 ± 2^a^*
	Pelota Control	4.47 ± 1.17^b^	6.39 ± 0.12^b^	143 ± 35^b^	6.02 ± 0.24^b^	135 ± 31^b^	0.76 ± 0.06^a^	17 ± 1^a^
	Pelota 350 MPa/5 min	6.70 ± 0.14^b^	7.40 ± 0.57^b^	110 ± 6^b^	8.49 ± 0.57^c^*	127 ± 1^b^	1.72 ± 0.14^a^*	26 ± 2^a^

^1^ mg/100 g fresh weight. Results are expressed as mean ± standard deviation (*n* = 3). Superscript lowercase letters indicate statistically significant differences (*p* ≤ 0.05) between digestive phases.* Indicate statistically significant differences (*p* ≤ 0.05) between treatments for the same variety.

**Table 5:** In vitro digestive stability (mg/100 g fresh weight) of betalains and phenolic compounds in HHP-treated (600 MPa/CUT) Sanguinos and Pelota prickly pear peels.

	Treatment	Peel	Oral Phase		Gastric Phase		Intestinal Phase	
		Content ^1^	Content ^1^	Recovery (%)	Content ^1^	Recovery (%)	Content ^1^	Recovery (%)
Indicaxanthin	Sanguinos Control	0.60 ± 0.01^b^	0.63 ± 0.03^b^	105 ± 3^b^	0.40 ± 0.01^a^	66 ± 1^a^	0.37 ± 0.02^a^	62 ± 4^a^
	Sanguinos 600 MPa/CUT	0.53 ± 0.03^a^	0.55 ± 0.08^a^	104 ± 8^a^	0.50 ± 0.16^a^	94 ± 25^a^	0.20 ± 0.03^a^*	39 ± 5^a*^
	Pelota Control	1.01 ± 0.01^c^	0.98 ± 0.06^c^	97 ± 5^c^	0.78 ± 0.07^b^	77 ± 6^b^	0.56 ± 0.03^a^	55 ± 3^a^
	Pelota 600 MPa/CUT	0.57 ± 0.06^b^*	0.65 ± 0.04^b^*	113 ± 5^b^	0.64 ± 0.07^b^	112 ± 0^b^*	0.39 ± 0.03^a^*	68 ± 5^a^
Betanin	Sanguinos Control	5.78 ± 0.23^c^	5.40 ± 0.50^c^	93 ± 5^c^	4.06 ± 0.46^b^	70 ± 5^b^	1.67 ± 0.13^a^	29 ± 2^a^
	Sanguinos 600 MPa/CUT	4.52 ± 0.28^b^*	5.18 ± 0.06^b^	115 ± 6^b^	5.00 ± 0.53^b^	111 ± 5^b^*	1.32 ± 0.11^a^*	29 ± 2^a^
	Pelota Control	16.29 ± 0.10^b^	17.73 ± 1.47^b^	109 ± 8^b^	15.90 ± 2.13^b^	98 ± 12^b^	7.51 ± 0.60^a^	46 ± 4^a^
	Pelota 600 MPa/CUT	13.92 ± 1.06^b^	13.20 ± 0.59^b^	95 ± 3^b^	12.50 ± 1.06^b^	90 ± 1^b^	5.11 ± 0.75^a^*	37 ± 3^a^
Piscidic acid	Sanguinos Control	439.45 ± 0.90^b^	463.24 ± 5.65^b^	105 ± 1^b^	401.20 ± 33.23^b^	91 ± 7^b^	230.10 ± 18.41^a^	52 ± 4^a^
	Sanguinos 600 MPa/CUT	346.50 ± 18.65^b^*	373.12 ± 8.66^b^*	108 ± 3^b^	454.47 ± 34.38^c^	131 ± 3^c^*	208.83 ± 16.70^a^	60 ± 5^a^
	Pelota Control	779.17 ± 4.74^b^	785.43 ± 40.00^b^	101 ± 4^b^	772.12 ± 36.45^b^	99 ± 4^b^	410.70 ± 32.86^a^	53 ± 4^a^
	Pelota 600 MPa/CUT	713.46 ± 59.11^b^	720.35 ± 39.82^b^	101 ± 3^b^	839.20 ± 49.96^b^	118 ± 3^c^*	364.88 ± 29.19^a^	51 ± 4^a^
4-hydroxy benzoic acid derivative	Sanguinos Control	19.12 ± 0.21^b^	20.57 ± 1.90^b^	108 ± 9^b^	17.45 ± 2.31^b^	91 ± 11^ab^	11.60 ± 0.93^a^	61 ± 5^a^
	Sanguinos 600 MPa/CUT	14.38 ± 0.73^a^*	16.47 ± 1.46^a^	114 ± 4^b^	17.61 ± 0.46^a^	122 ± 3^ab^	15.40 ± 1.23^a^	107 ± 9^a^*
	Pelota Control	23.62 ± 0.14^ab^	27.44 ± 3.71^b^	116 ± 15^a^	25.33 ± 3.75^ab^	107 ± 15^a^	15.48 ± 1.24^a^	66 ± 5^a^
	Pelota 600 MPa/CUT	25.67 ± 4.28^a^	32.74 ± 1.64^ab^	128 ± 15^b^	41.83 ± 5.00^b^	163 ± 8^b^*	21.10 ± 2.20^a^	82 ± 9^a^
IG1 ^2^	Sanguinos Control	0.55 ± 0.03^ab^	0.63 ± 0.09^b^	115 ± 11^b^	0.85 ± 0.13^b^	156 ± 17^b^	0.31 ± 0.03^a^	56 ± 5^a^
	Sanguinos 600 MPa/CUT	0.67 ± 0.06^a^	0.74 ± 0.26^a^	111 ± 29^a^	0.81 ± 0.24^a^	122 ± 25^a^	0.46 ± 0.04^a^*	69 ± 6^a^*
	Pelota Control	1.44 ± 0.01^b^	1.55 ± 0.15^b^	108 ± 10^b^	1.45 ± 0.23^b^	101 ± 15^b^	0.77 ± 0.06^a^	53 ± 4^a^
	Pelota 600 MPa/CUT	2.79 ± 0.17^b^*	2.74 ± 0.09^b^*	98 ± 3^b^*	2.72 ± 0.14^b^*	97 ± 1^b^*	1.73 ± 0.14^a^*	62 ± 5^a^*
IG2 ^3^	Sanguinos Control	1.51 ± 0.13^b^	1.47 ± 0.14^b^	98 ± 1^c^	0.87 ± 0.10^a^	58 ± 2^b^	0.63 ± 0.05^a^	42 ± 3^a^
	Sanguinos 600 MPa/CUT	1.50 ± 0.06^b^	1.61 ± 0.18^b^*	107 ± 8^b^	1.60 ± 0.13^b^*	107 ± 5^b^*	0.89 ± 0.07^a^	59 ± 5^a^*
	Pelota Control	0.35 ± 0.00^a^	0.45 ± 0.05^a^	130 ± 14^a^	0.42 ± 0.09^a^	121 ± 26^a^	0.25 ± 0.02^a^	73 ± 6^a^
	Pelota 600 MPa/CUT	0.64 ± 0.05^b^*	0.56 ± 0.01^b^	89 ± 6^b^	0.55 ± 0.01^b^	87 ± 5^b^	0.35 ± 0.03^a^	55 ± 4^a^
IG3 ^4^	Sanguinos Control	0.32 ± 0.03^b^	0.33 ± 0.04^b^	103 ± 2^c^	0.26 ± 0.03^b^	83 ± 2^b^	0.15 ± 0.01^a^	48 ± 3^a^
	Sanguinos 600 MPa/CUT	0.32 ± 0.01^b^	0.33 ± 0.03^b^	105 ± 6^b^	0.30 ± 0.02^b^	96 ± 3^b^*	0.20 ± 0.02^a^*	63 ± 5^a^*
	Pelota Control	0.15 ± 0.00^ab^	0.18 ± 0.00^b^	125 ± 5^b^	0.18 ± 0.03^b^	120 ± 26^b^	0.08 ± 0.01^a^	57 ± 4^a^
	Pelota 600 MPa/CUT	0.23 ± 0.01^c^	0.20 ± 0.01^b^	85 ± 1^b^*	0.19 ± 0.00^b^	80 ± 1^b^	0.14 ± 0.1^a^*	59 ± 5^a^
IG4 ^5^	Sanguinos Control	0.81 ± 0.06^b^	0.73 ± 0.04^b^	89 ± 2^b^	0.53 ± 0.06^a^	65 ± 3^a^	0.49 ± 0.04^a^	60 ± 5^a^
	Sanguinos 600 MPa/CUT	0.71 ± 0.03^a^	0.83 ± 0.05^a^	116 ± 1^a^*	0.85 ± 0.19^a^	120 ± 21^a^	0.56 ± 0.04^a^	79 ± 6^a^*
	Pelota Control	0.23 ± 0.00^a^	0.24 ± 0.03^a^	104 ± 14^a^	0.23 ± 0.10^a^	97 ± 41^a^	0.14 ± 0.01^a^	59 ± 4^a^
	Pelota 600 MPa/CUT	0.44 ± 0.04^c^*	0.38 ± 0.00^bc^*	85 ± 6^b^	0.32 ± 0.01^b^	72 ± 5^b^	0.18 ± 0.01^a^	40 ± 3^a^*
IG5 ^6^	Sanguinos Control	3.09 ± 0.18^c^	2.89 ± 0.07^bc^	93 ± 3^c^	2.56 ± 0.08^b^	83 ± 2^b^	0.92 ± 0.07^a^	30 ± 2^a^
	Sanguinos 600 MPa/CUT	2.82 ± 0.11^b^	2.57 ± 0.11^b^	91 ± 2^b^	2.49 ± 0.42^b^	88 ± 11^b^	1.30 ± 0.11^a^*	46 ± 4^a^*
	Pelota Control	2.26 ± 0.01^b^	2.29 ± 0.21^b^	101 ± 9^b^	2.26 ± 0.19^b^	100 ± 8^b^	1.26 ± 0.10^a^	56 ± 4^a^
	Pelota 600 MPa/CUT	5.10 ± 0.49^c^*	4.78 ± 0.07^bc^*	94 ± 8^b^	3.91 ± 0.09^b^*	77 ± 5^b^	2.21 ± 0.18^a^*	43 ± 4^a^*

Results are expressed as mean ± standard deviation (*n* = 3). Superscript lowercase letters indicate statistically significant differences (*p* ≤ 0.05) between digestive phases. * Indicate statistically significant differences (*p* ≤ 0.05) between treatments for the same variety. ^1^ mg/100 g fresh weight, ^2^ isorhamnetin glucosyl-rhamnosyl-rhamnoside (IG1), ^3^ isorhamnetin glucosyl-rhamnosyl-pentoside (IG2), ^4^ isorhamnetin hexosyl-hexosyl-pentoside (IG3), ^5^ isorhamnetin glucosyl-pentoside (IG4), and ^6^ isorhamnetin glucosyl-rhamnoside (IG5).

## Supplementary Materials

The following are available online, Figure S1: Bioaccessibility (%) of (A) indicaxanthin, (B) betanin, (C), piscidic acid, and (D) 4-hydroxybenzoic acid glycoside in Sanguinos and Pelota prickly pear pulps treated with HHP (100, 350, and 600 MPa; CUT and 5 min); Figure S2: Bioaccessibility (%) of (A) indicaxanthin, (B) betanin, (C) piscidic acid, and (D) 4-hydroxybenzoic acid glycoside, (E) IG1, (F) IG2, (G) IG3, (H) IG4 and (I) IG5 in Sanguinos and Pelota prickly pear peels treated with HHP (100, 350 and 600 MPa; CUT and 5 min); Figure S3: Content (mg/100 g fresh pulp) of (A) indicaxanthin, (B) betanin, (C) piscidic acid, and (D) 4-hydroxybenzoic acid glycoside in Sanguinos and Pelota prickly pear pulps treated with HHP (350 MPa/5 min) and submitted to in vitro simulated gastrointestinal digestion (oral phase, gastric phase, intestinal phase); Figure S4: Content (mg/100 g fresh peel) of (A) indicaxanthin, (B) betanin, (C) piscidic acid (D) 4-hydroxybenzoic acid glycoside, (E) IG1, (F) IG2, (G) IG3, (H) IG4 and (I) IG5 in Sanguinos and Pelota prickly pear peels treated with HHP (600 MPa/CUT) and submitted to in vitro simulated gastrointestinal digestion (oral phase, gastric phase, intestinal phase).

## Sample Availability

Samples of the compounds are not available from the authors.
