# Supplementary figures and images for: Bioaccessibility of Antioxidants in Prickly Pear Fruits Treated with High Hydrostatic Pressure: An Application for Healthier Foods
Source: Molecules. 2021 Aug 30;26(17):5252. doi: 10.3390/molecules26175252 (PMC8434551; doi:10.3390/molecules26175252)

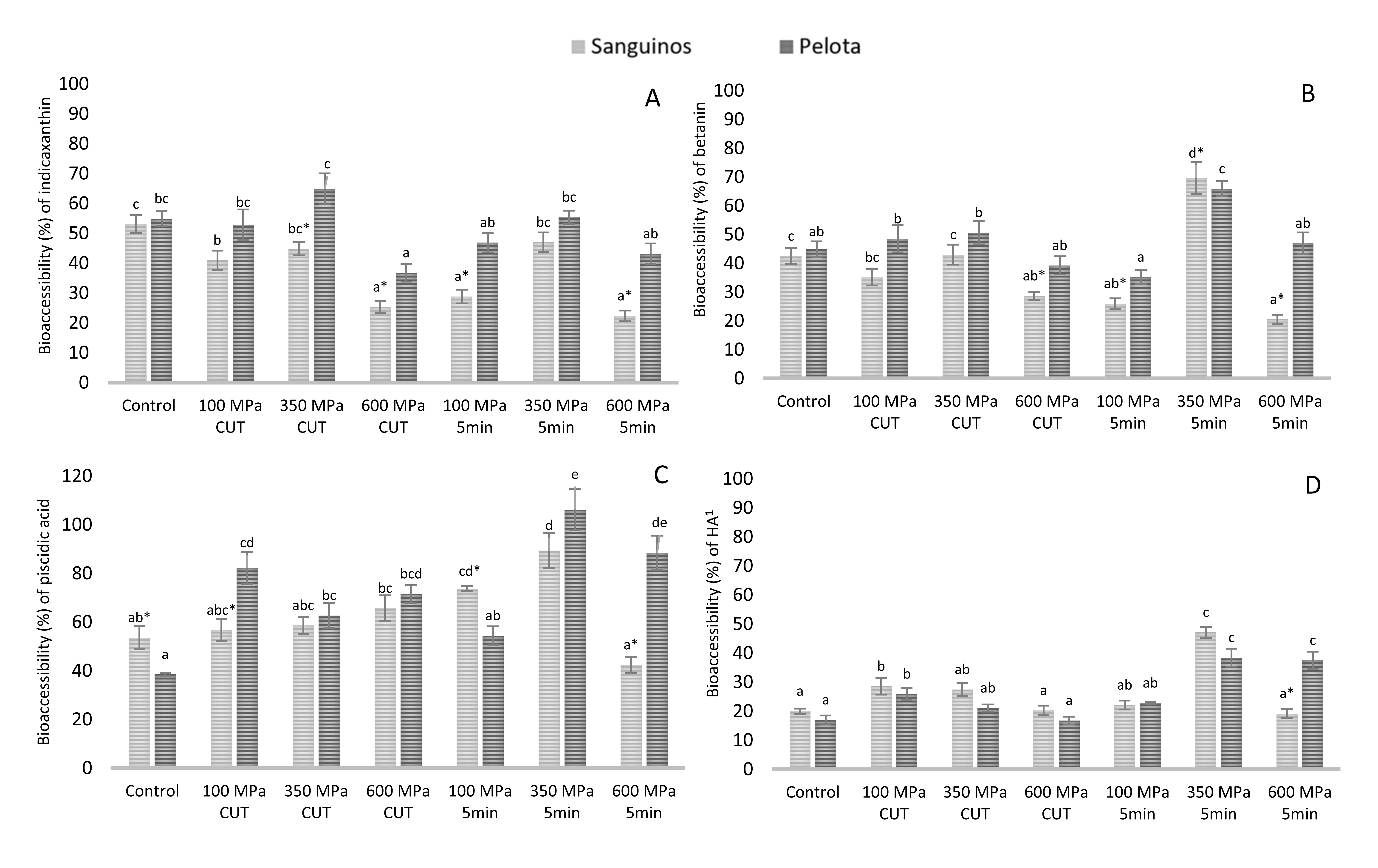

Supplement: Supplementary file 1 [file molecules-26-05252-s001.zip › molecules-1327137-supplementary/Supplementary Figure S1.tif]

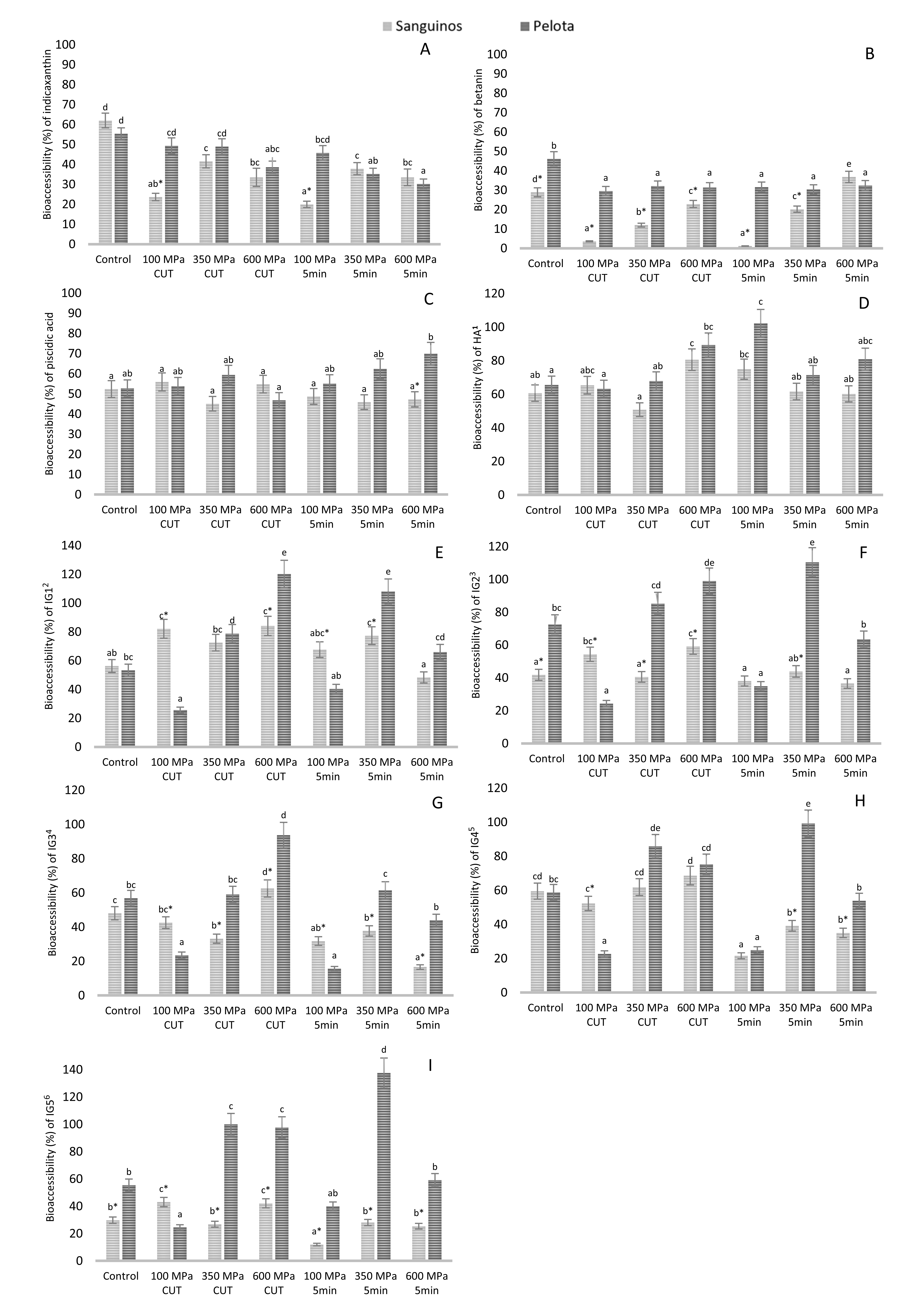

Supplement: Supplementary file 1 [file molecules-26-05252-s001.zip › molecules-1327137-supplementary/Supplementary Figure S2.tif]

◆ Sanguinos    ■ Sanguinos 600 MPa/CUT    ▲ Pelota    ✕ Pelota 600 MPa/CUT

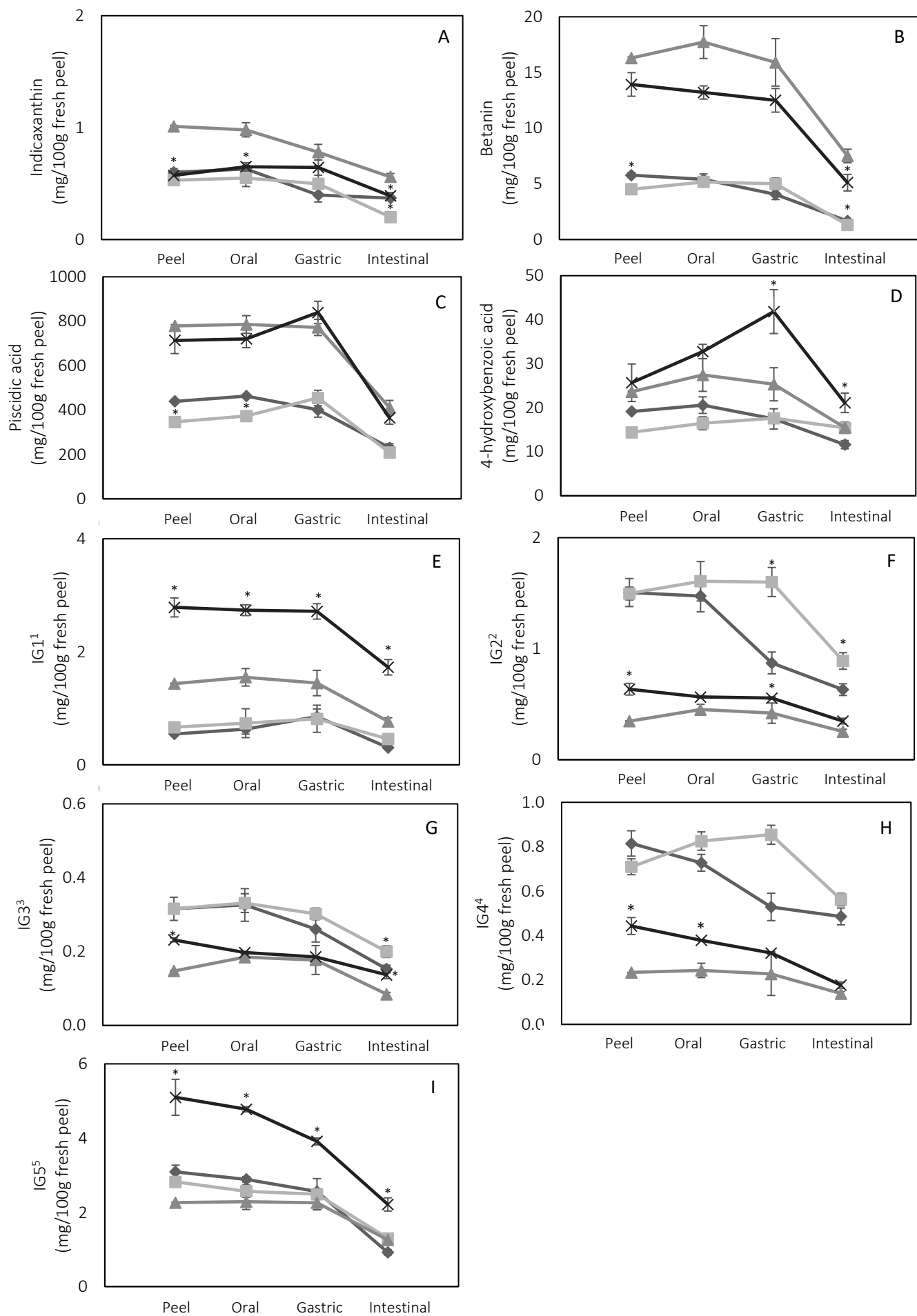

Supplement: Supplementary file 1 [file molecules-26-05252-s001.zip › molecules-1327137-supplementary/Supplementary Figure S3.pdf]

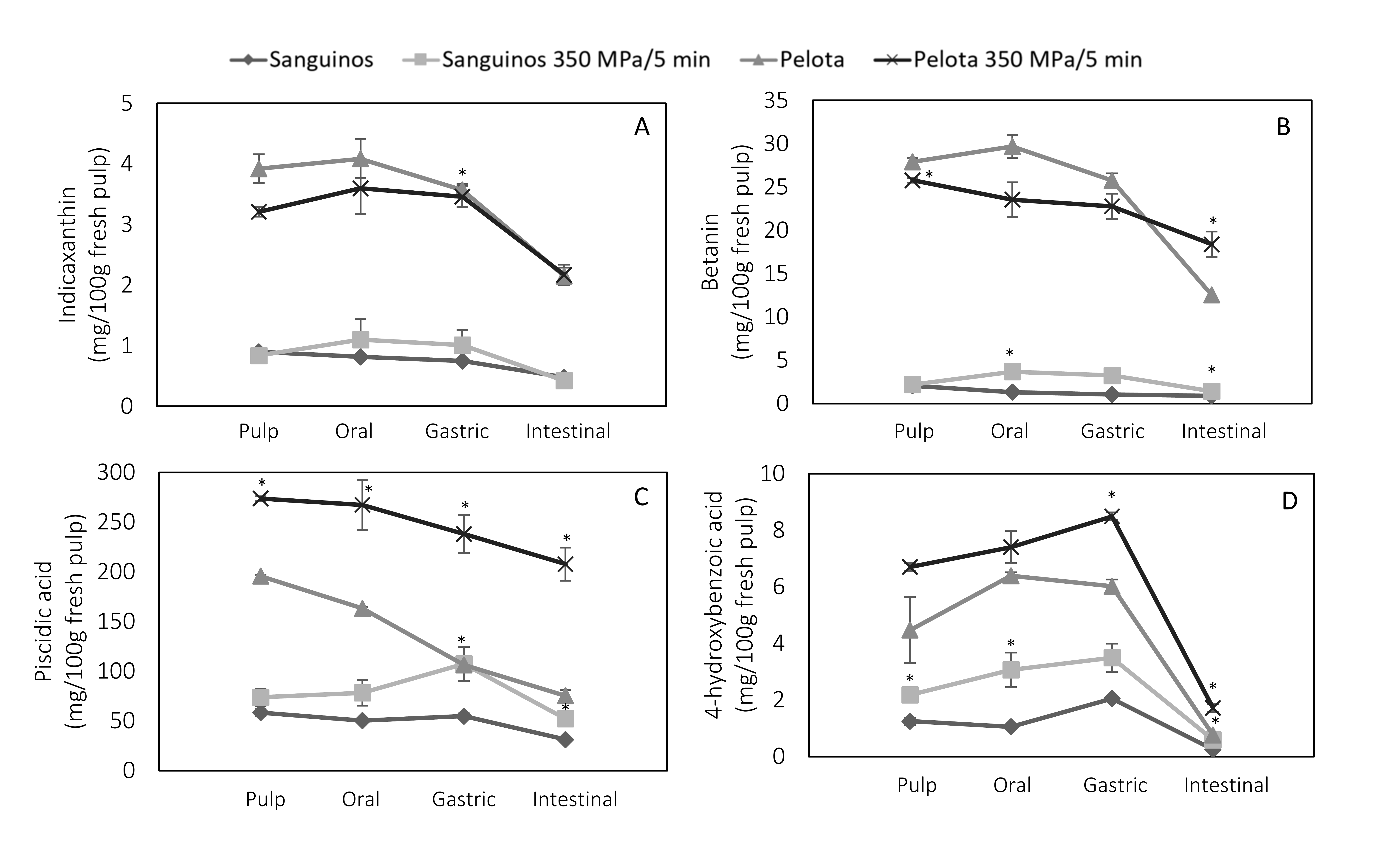

Supplement: Supplementary file 1 [file molecules-26-05252-s001.zip › molecules-1327137-supplementary/Supplementary Figure S3.tif]
